# The alga *Euglena gracilis* stimulates *Faecalibacterium* in the gut and contributes to increased defecation

**DOI:** 10.1038/s41598-020-80306-0

**Published:** 2021-01-13

**Authors:** Ayaka Nakashima, Kengo Sasaki, Daisuke Sasaki, Kosuke Yasuda, Kengo Suzuki, Akihiko Kondo

**Affiliations:** 1 The Research and Development Department, euglena Co., Ltd., Tokyo, 108-0014 Japan; 2 Graduate School of Science, Technology, and Innovation, Kobe University, Kobe, Hyogo 657-8501 Japan; 3 RIKEN Center for Sustainable Resource Science, Yokohama, Kanagawa 230-0045 Japan

**Keywords:** Biomarkers, Health care

## Abstract

The alga *Euglena gracilis* (*E. gracilis*) has recently gained attention as a health food, but its effects on human gut microbiota remain unknown. This study aimed to determine the effect of *E. gracilis* on gut microbiota and defecation due to modulation of microbiota composition in vitro and in vivo. The in vitro model simulating human colonic microbiota revealed that *E. gracilis* addition stimulated the growth of commensal *Faecalibacterium*. Further, *E. gracilis* addition enhanced butyrate production by *Faecalibacterium prausnitzii*. Paramylon, an insoluble dietary fibre that accumulates in *E. gracilis* and is the main component of *E. gracilis*, did not stimulate *Faecalibacterium* growth in vitro. Daily ingestion of 2 g of *E. gracilis* for 30 days increased bowel movement frequency as well as stool volume in 28 human participants. Collectively, these findings indicate that *E. gracilis* components other than paramylon, stimulate the growth of *Faecalibacterium* to improve digestive health as well as promote defecation by increasing butyrate production.

## Introduction

Photosynthetic microalgae are present in both marine and freshwater environments and have attracted attention owing to their potential applications in nutraceuticals^1^. Microalgae produce various compounds that can be consumed as food products. These compounds are not only used to supplement the diet but are also used in the prevention or treatment of diseases and/or disorders^1,2^. *Euglena gracilis* (*E. gracilis*) is a candidate fast-proliferating microalga, besides *Chlorella* and *Spirulina*, and research into its commercial cultivation is ongoing^3^. *E. gracilis* is a rich source of vitamins, minerals, unsaturated fatty acids, and a crystalline β-1,3-glucan polysaccharide called paramylon, which is considered a functional dietary fibre^4,5^. *E. gracilis* is often used as a dietary supplement. In recent years, the immunomodulatory effects of *E. gracilis* have been reported in both humans and animal models^6^. *E. gracilis* supplementation improved hyperglycemia in a type 2 diabetes mellitus rat model^7^ and provided protection against influenza virus infection in mice^8^. Furthermore, oral administration of paramylon relieved the symptoms of atopic dermatitis in mice^9^, exerted hepatoprotective effects via antioxidative action in rats^10^, and exerted preventive effects against colon cancer in mice^11^.

The gastrointestinal tract is colonised by a complex bacterial community, the intestinal microbiota, which interacts with the host and exerts a strong impact on host homeostasis and immunostasis. Therefore, intestinal microbiota is essential for maintaining host health^12–14^. Despite an explosion of interest in optimising the composition of intestinal microbiota by dietary means using probiotics^15^ or functional food products^16^, the effect of *E. gracilis* ingestion on human gut microbiota remains unknown.

The gut microbiota-modulating effects of diet are often investigated via in vivo studies in humans^17^. Information on the composition of colonic microbiota originates mainly from the analysis of faecal samples in human dietary intervention studies. However, this method is limited by the fact that the production of metabolites, such as short-chain fatty acids (SCFAs), cannot be measured at the actual site (the intestinal tract). An in vitro study method using a model culture system was previously developed in our laboratory to closely reproduce the microbial components in human faecal inoculum^18^. This in vitro human colonic microbiota model detected the decreased production of butyrate in ulcerative colitis patients^18^. Thus, combining in vitro and in vivo studies can help interpret the changes in human gut microbiota.

This study aimed to evaluate the effect of *E. gracilis* consumption on human colonic microbiota in healthy human subjects and validate the findings by analysing effects of adding *E. gracilis* or paramylon to an in vitro human colonic microbiota model.

## Results

### *Euglena gracilis* addition changed microbiota composition and enhanced butyrate production by *Faecalibacterium *in in vitro model culture system

**Figure 1:**
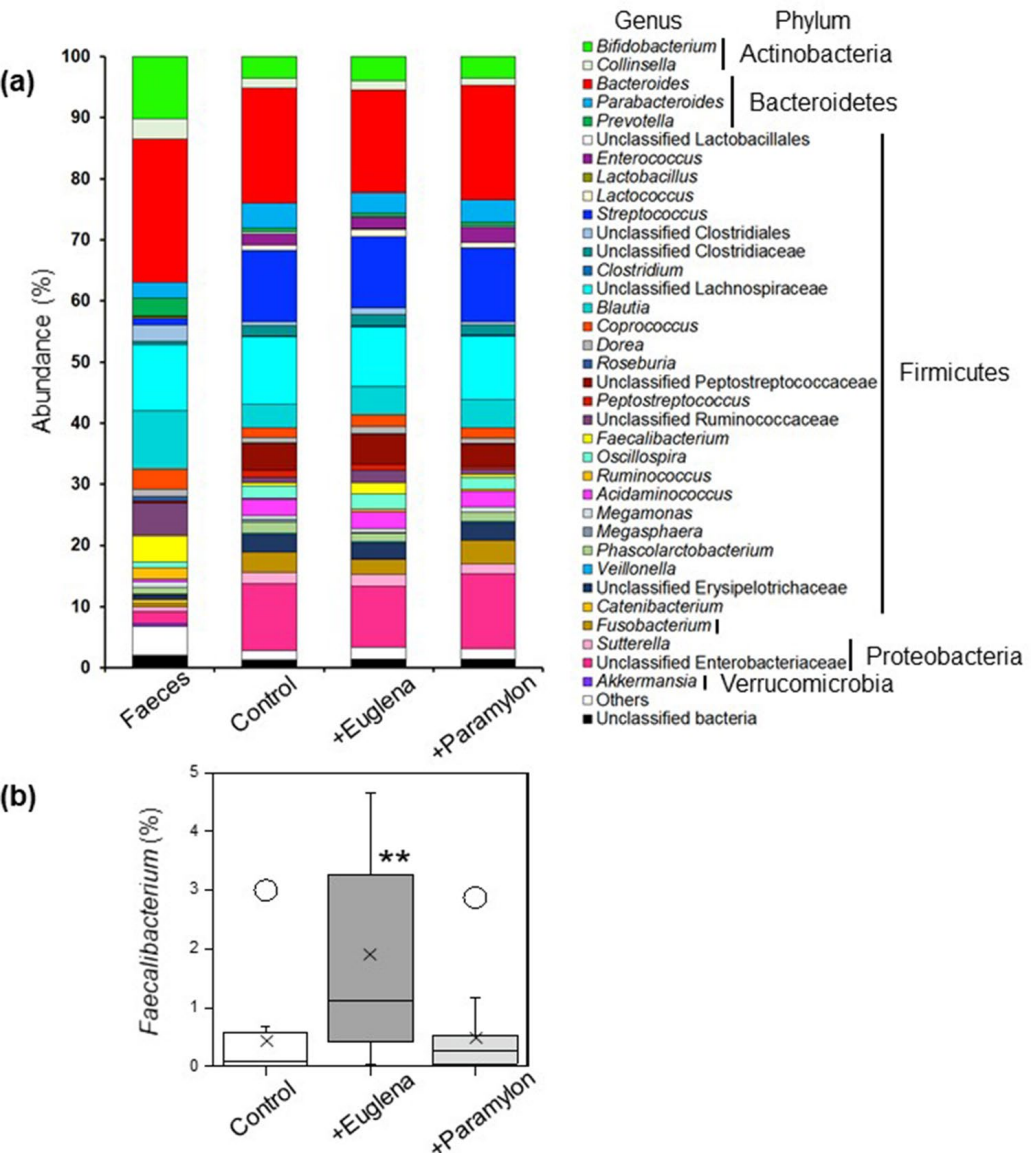
*Euglena gracilis* addition changes microbiota composition. (**a**) Genus-level compositional view of bacteria in the original faecal samples (Faeces), in vitro human colonic microbiota models (Control), models with *E. gracilis* (+ Euglena), and models with paramylon (+ Paramylon) after 48 h of fermentation. The means of 11 samples from healthy human subjects are shown. Genera of lower abundance (< 1.0%) and lower similarity (< 97%) were included in Others and Unclassified Bacteria, respectively. (**b**) Box-and-whisker plots representing relative abundances of bacteria related to *Faecalibacterium*. **P < 0.01, n = 11, Wilcoxon signed rank test. Circles outside the whiskers represent outlier samples.

Faecal samples were obtained from 11 healthy subjects. Each sample was used as an inoculum and cultivated for 48 h to construct an in vitro human colonic microbiota model. Three microbiota models were constructed for each subject (without addition [Control], with 9 g/L *E. gracilis* [+Euglena], and with 3 g/L paramylon [+Paramylon]). Total bacterial DNA was extracted from the original faecal samples and the three corresponding groups (Control, +Euglena, and +Paramylon). Sequencing of bacterial 16S rRNA genes provided 8,699,439 reads (Supplementary Table S1). The corresponding in vitro models maintained the bacterial species richness of the original faeces; the Chao1 index showed no difference between faecal inoculums and corresponding in vitro models (*P* = 0.69, Mann–Whitney *U* test). Based on Shannon index data, bacterial diversity was slightly lower in the corresponding in vitro models than in the original faecal samples (*P* = 0.022, Mann–Whitney *U* test). Inverse Simpson scores showed a similar trend between faecal inoculums and corresponding in vitro models (*P* = 0.076, Mann–Whitney *U* test). Genus level bacterial composition was compared between corresponding in vitro models (Fig. 1a). The microbial composition of in vitro models with added *E. gracilis* or paramylon (+Euglena, or +Paramylon) was similar to that of the control model. However, in the in vitro model, *E. gracilis* addition (+Euglena) resulted in a significant increase (average 1.92%) in the relative abundance of *Faecalibacterium* bacteria than in the control conditions (average 0.47%) (*P* = 0.0038, paired Wilcoxon signed rank test; Fig. 1b). In contrast, compared with the control, paramylon addition (+Paramylon) did not change the relative abundance (average 0.54%) of *Faecalibacterium* in the in vitro model (*P* = 0.54, paired Wilcoxon signed rank test; Fig. 1b).

**Figure 2:**
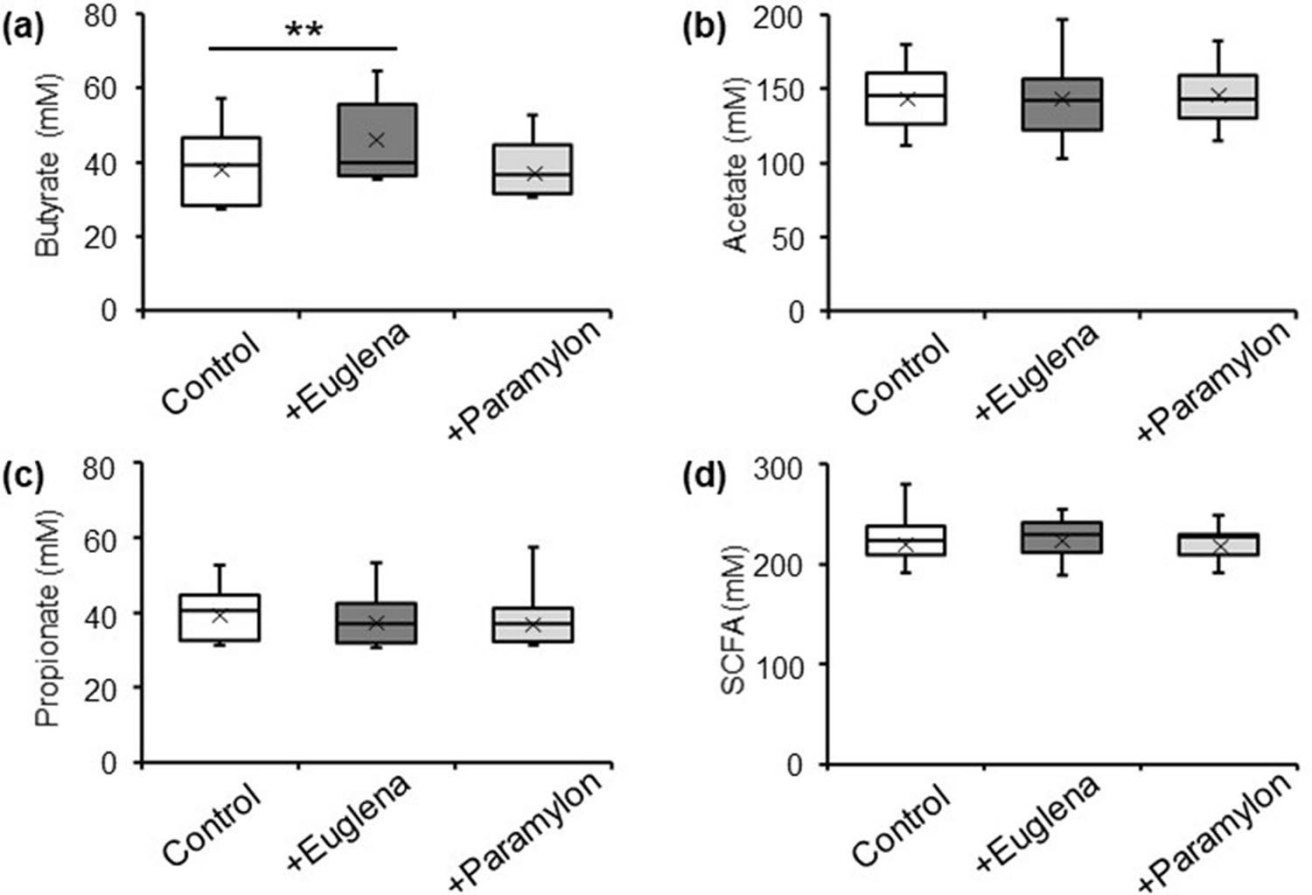
*Euglena gracilis* addition enhanced butyrate concentrations. Production of (**a**) butyrate, (**b**) acetate, (**c**) propionate, and (**d**) short-chain fatty acids (SCFAs) (sum of lactate, succinate, acetate, propionate, and butyrate) in in vitro human colonic microbiota models (Control), models with *E. gracilis* (+ Euglena), and models with paramylon (+ Paramylon) after 48 h fermentation. **P < 0.01, n = 11, Wilcoxon signed rank test.

The SCFA production in the in vitro microbiota models (Control, +Euglena, and +Paramylon) was measured after 48 h of fermentation. The average butyrate production in *E. gracilis* addition (+Euglena) was 45.13 mM compared with 38.65 mM in the control. *E. gracilis* addition (9 g/L) significantly increased the butyrate production (*P* = 0.0098, paired Wilcoxon signed rank test), although no significant changes in acetate or propionate production were detected between in vitro models without or with *E. gracilis* (Control or +Euglena; *P* = 0.97 and 0.10, respectively, paired Wilcoxon signed rank test; Fig. 2). Paramylon addition (3 g/L) did not change the production of acetate, propionate, or butyrate (*P* = 0.52, 0.17, and 0.64, respectively, paired Wilcoxon signed rank test).

### *Euglena gracilis* addition increased the growth of *Faecalibacterium prausnitzii* and enhanced butyrate production by *Faecalibacterium prausnitzii* in vitro

**Figure 3:**
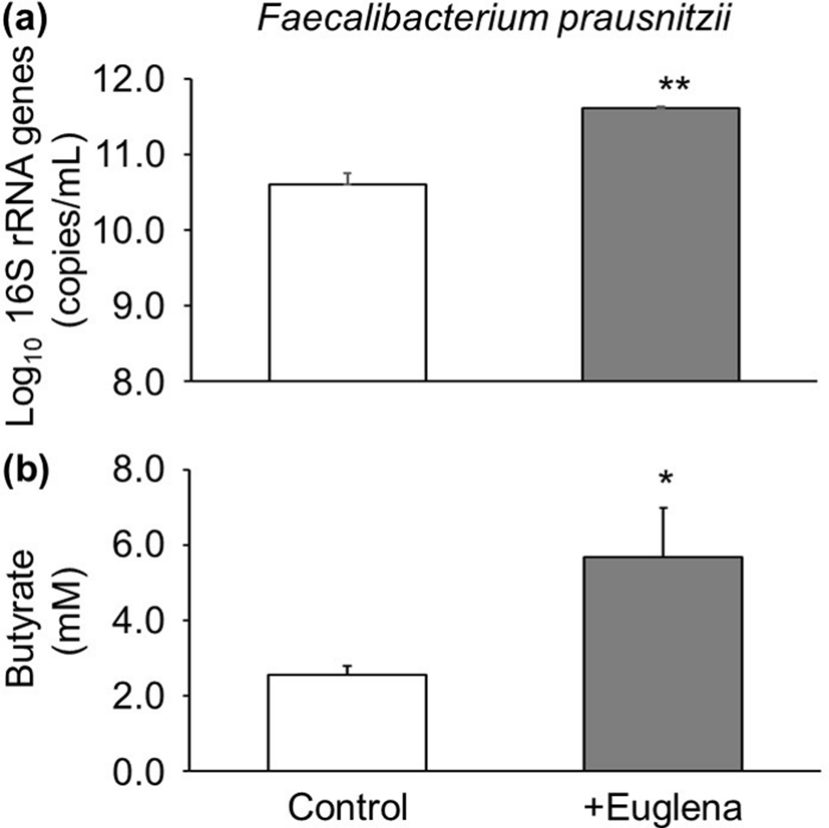
(**a**) Cells detected by real-time PCR and (**b**) concentration of butyrate produced by *Faecalibacterium prausnitzii* without *Euglena gracilis* (Control) and with *E. gracilis* addition (+ Euglena) after 72 h of cultivation. *P < 0.05, **P < 0.01, n = 3, two-tailed Student’s *t*-test.

We investigated the effect of *E. gracilis* addition (9 g/L) on a single species, *Faecalibacterium prausnitzii* (*F. prausnitzii*). Real time PCR assay showed that *E. gracilis* addition increased the growth of *F. prausnitzii* (average 11.61 ± 10.34 copies/mL) at 72 h of culture compared with an average of 10.61 ± 10.22 copies/mL in samples without *E. gracilis* (*P* < 0.0001, two-tailed Student’s *t*-test; Fig. 3a). At the same time, *E. gracilis* addition increased the butyrate production by *F. prausnitzii* to an average of 5.67 ± 1.30 mM compared with 2.55 ± 0.23 mM in samples without *E. gracilis* (Control) (*P* = 0.015, two-tailed Student’s *t*-test; Fig. 3b).

### *Euglena gracilis* ingestion increased the relative abundance of *Faecalibacterium* in human gut flora and promoted defecation

**Figure 4:**
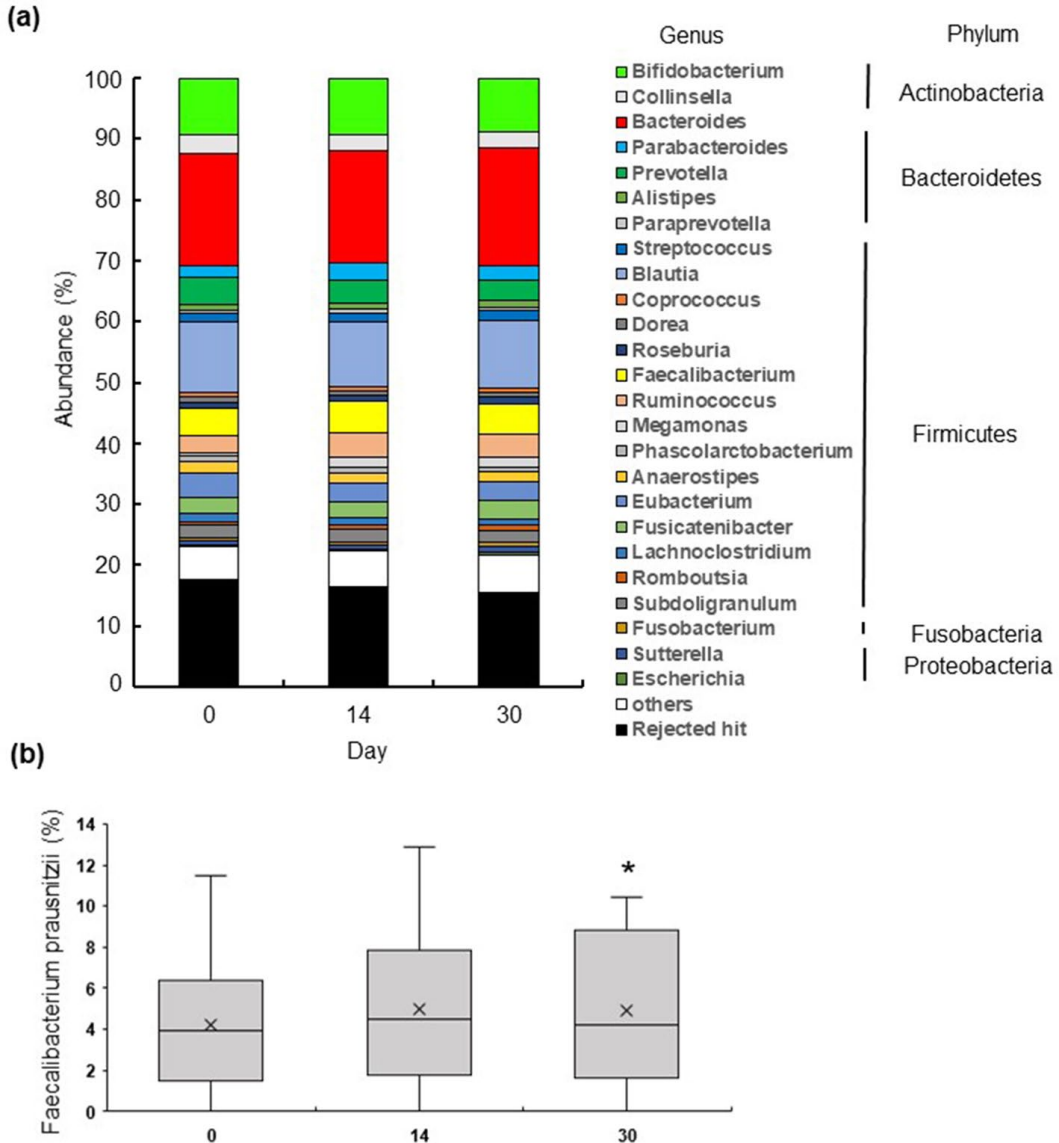
*Euglena gracilis* ingestion increases the proportion of the *Faecalibacterium* genus in the gut flora. (**a**) Genus-level compositional view of bacteria in human faecal samples after 0, 14, and 30 days of *E. gracilis* ingestion. (**b**) The relative abundances of the *Faecalibacterium* genus. *P < 0.05, n = 28, Wilcoxon signed rank test.

We further examined the changes in the intestinal microflora before and after continuous *E. gracilis* ingestion in 28 human participants (Fig. 4a). Sequencing of bacterial 16S rRNA provided 3,047,422 reads (Supplementary Table S2). Importantly, 30 days after the beginning of the ingestion of *E. gracilis* (2 g/day) the bacterial species richness was maintained (vs. the baseline); the Chao1 and Shannon indexes and the Inverse Simpson score were not significantly different between days 0 and 30 (*P* = 0.76, 0.84 and 0.45, respectively; Kruskal–Wallis test). Comparison of faecal samples collected before (0 days) and at 14 and 30 days after ingesting *E. gracilis* (2 g/day) showed that the relative abundance of bacteria related to the *Faecalibacterium* genus was unchanged after 14 days (*P* = 0.18, paired Wilcoxon signed rank test). However, the relative abundance of *Faecalibacterium* upon *E. gracilis* ingestion (average 5.10%) significantly increased after 30 days compared with that before *E. gracilis* ingestion (0 days; average 4.35%) (*P* = 0.04, paired Wilcoxon signed rank test; Fig. 4b).

**Figure 5:**
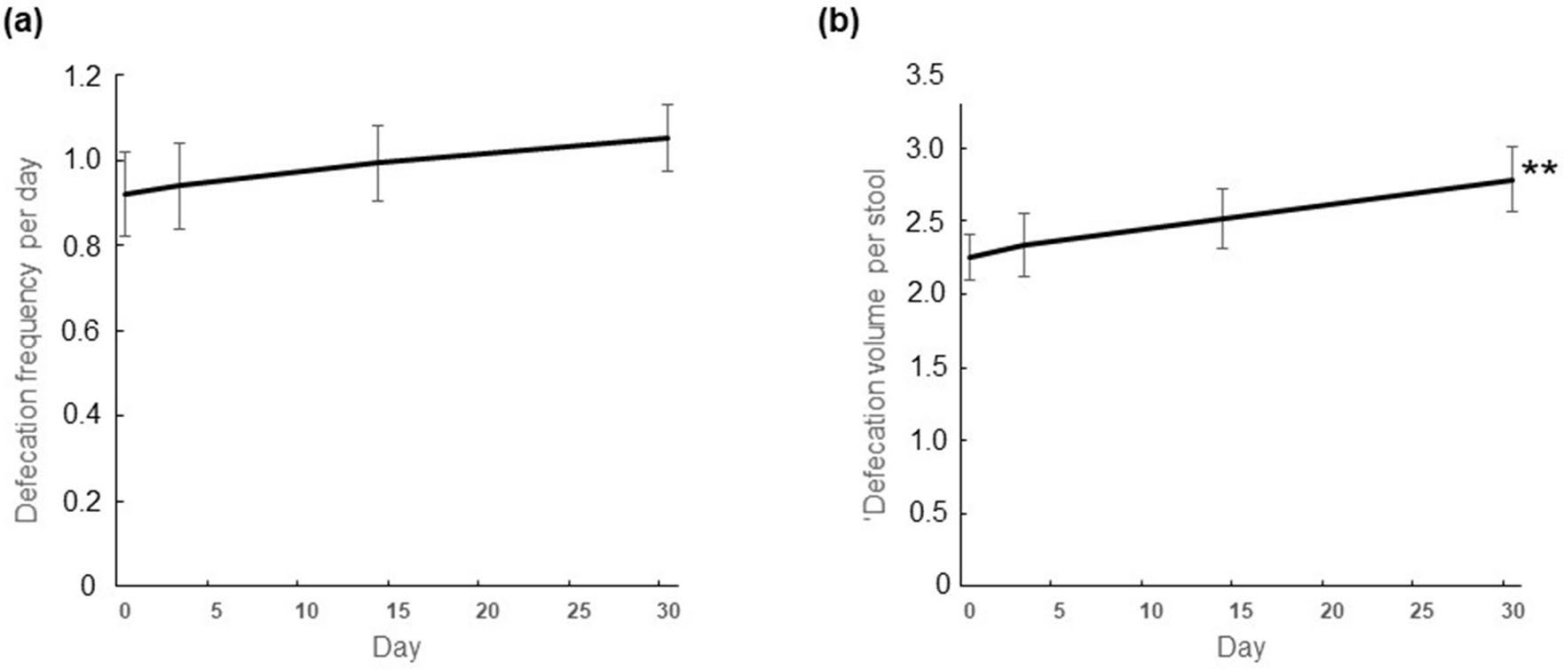
*Euglena gracilis* ingestion promotes defecation. (**a**) The frequency of defecation per day and (**b**) the volume of defecation per stool. One defecation volume is equivalent to one medium-sized chicken egg (about 60 g). Mean ± SEM, **P < 0.01, n = 28, Wilcoxon signed rank test.

We simultaneously examined the changes in daily defecation quantity before and after daily ingestion of *E. gracilis* (2 g/day) in 28 human participants using the Bristol Stool Form Scale^19^. All outcomes were compared before and after *E. gracilis* ingestion. The number of bowel movements per day increased from an average of 0.92 ± 0.10 to an average of 1.05 ± 0.08 (*P* = 0.069, paired Wilcoxon signed rank test, Fig. 5a) and the amount of stool per bowel movement increased significantly from an average of 2.25 ± 0.16 to an average of 2.79 ± 0.23 (*P* = 0.009, paired Wilcoxon signed rank test, Fig. 5b), respectively.

## Discussion

This study investigated the effect of *E. gracilis* consumption on human gut microbiota and defecation characteristics in human subjects. Both in vitro and in vivo experiments revealed that *E. gracilis* consumption significantly increased the relative abundance of bacteria belonging to the *Faecalibacterium* genus. However, this effect did not appear to be due to paramylon consumption. *F. prausnitzii*, the only known species in the genus *Faecalibacterium*, is one of the most abundant butyrate-producing bacteria in the gastrointestinal tract^20^. Compared with fermentation without *E. gracilis,* butyrate production increased after fermenting with *E. gracilis* due to the increase in *Faecalibacterium*. At this time, there was no difference in other butyric acid producing bacteria, such as *Roseburia*, both in vitro and in vivo. Thus, the changes in the flora caused by *E. gracilis* may be specific to *Faecalibacterium*. Previous studies reported that butyrate improves insulin resistance^21^ and that increasing the abundance of butyrate-producing bacteria may be a feasible strategy to counteract type 2 diabetes^22^. Our results corroborate with those of a study, which showed that *E. gracilis* supplementation decreased blood glucose concentration in a type 2 diabetes mellitus rat model^7^. It was shown that components in *E. gracilis* other than paramylon enhanced *Faecalibacterium*. *E. gracilis* is also a source of vitamins, minerals, and unsaturated fatty acids^4,5^; these components would promote the growth of *Faecalibacterium*. Another possibility is that *E. gracilis* indirectly stimulates *Faecalibacterium*. Our previous study showed that *E. gracilis* consumption increases *Bifidobacterium* in the gut microbiota of mice^23^. Here, the relative abundance of *Bifidobacterium* did not significantly increase in vitro or in vivo by *E. gracilis* consumption (*P* = 0.46 and 0.65, respectively, paired Wilcoxon signed rank test; Figs. 1 and 4). Thus, *E. gracilis* consumption may stimulate acetate production by *Bifidobacterium*, which in turn, could be consumed by *Faecalibacterium* to enhance its growth and/or butyrate formation. A cross-feeding process between *Bifidobacterium adolescentis* and *F. prausnitzii* was previously reported using fructooligosaccharide as a carbon source^24^.

Further, stool volume increased in participants who ingested *E. gracilis* compared with those who consumed non-supplemented food. Our previous results have also shown increased stool volumes in individuals who consumed *E. gracilis*^25^. These findings were also consistent with those of Kawano et al., who found that cholesterol residence time in the gut was shorter in rats fed a diet containing cholesterol and *E. gracilis* compared with that in rats fed a diet containing cholesterol alone^26^.

Dietary fibre intake can increase stool frequency in patients with constipation^27^. Paramylon in *E. gracilis* is insoluble and not digested or absorbed by the body; thus, it is believed to exert effects similar to those of dietary fibre. In addition, increased butyrogenesis via *F. prausnitzii* enhancement caused by *E. gracilis* consumption corresponded with that in a previous study, which showed butyrate supplementation potentially reduces difficulties in bowel movement^28^. Thus, *E. gracilis* consumption could have a beneficial effect on constipation, such as in reducing pain during defecation. Further, butyrate produced in the gut increases the number of specific CD8+ cells that eliminate the influenza virus and control infection^29^. These findings are consistent with studies, which have shown that *E. gracilis* supplementation regulates immunity^6,8^, and that the contribution of *E. gracilis* to immunity may not be because of paramylon alone.

In summary, our work shows that consuming *E. gracilis* stimulates *Faecalibacterium* growth and butyrate production. This could be a mechanism to improve stool volume. One of the limitations of this study is the small sample size in the in vivo analysis. Therefore, it is necessary to increase the sample size in future studies to validate the findings of the present study. Further experiments are also needed to identify components other than paramylon in *E. gracilis*. Nevertheless, this study demonstrates the potential use of *E. gracilis* as a novel prebiotic.

## Methods

### *Euglena gracilis* preparation

*E. gracilis* was prepared at euglena Co., Ltd. (Tokyo, Japan). Components of *E. gracilis* were measured as described previously^7^. *E. gracilis* is composed of 52.2% carbohydrates, 28.6% protein, and 13.0% fat. Approximately 70–80% of the carbohydrates in *E. gracilis* was paramylon. Paramylon was isolated as described previously^30^. *E. gracilis* and paramylon used in this study were dry powders.

### Faecal collection and model culture system operation

Fresh faecal samples were obtained from 11 healthy human volunteers who had no history of antibiotic treatment for more than six months and provided written informed consent prior to sample collection. Immediately following collection, each faecal sample was stored using an anaerobic culture swab system (212550 BD BBL Culture Swab, Becton, Dickinson and Company, Franklin Lakes, NJ, USA) and used within 24 h. All experimental protocols were approved by the Institutional Ethics Review Board of Kobe University and in accordance with the guidelines approved by the Medical Ethics Committee at Kobe University.

The model culture system used a multichannel fermenter (Bio Jr. 8; ABLE, Tokyo, Japan) as previously described^18^. Inoculum was prepared by suspending each faecal sample in phosphate buffer (2 mL, 0.1 M, pH 6.5, consisting of a 68.5:31.5 mixture of 0.1 M NaH_2_PO_4_ and 0.1 M Na_2_HPO_4_) supplemented with 1% l-ascorbic acid (Wako Pure Chemical Industries, Osaka, Japan). Fermentation was initiated by the inoculation of autoclaved Gifu anaerobic medium (Nissui Pharmaceutical Co., Tokyo, Japan) with the faecal suspension (100 μL). We prepared three types of media for each human faecal inoculum, medium without addition (Control), medium containing *E. gracilis* (9 g/L) (+Euglena), and medium containing paramylon (3 g/L) (+Paramylon). Aliquots of fermentation cultures were sampled through the side projection of the vessel. Faecal samples and fermentation cultures were stored at − 20 °C until further use. The concentrations of SCFAs (acetate, propionate, and butyrate) were measured using a high-performance liquid chromatography (HPLC) system (Shimadzu, Kyoto, Japan) equipped with an Aminex HPX-87H column (Bio-Rad Laboratories, Hercules, CA, USA) and a RID-10A refractive index detector (Shimadzu).

### *Faecalibacterium* strain culture

*Faecalibacterium prausnitzii* JCM31915 was obtained from the Japan Collection of Microorganisms. *F. prausnitzii* was cultured in autoclaved Gifu anaerobic medium at 37 °C under anaerobic conditions (10% H_2_, 10% CO_2_, and 80% N_2_) for three days. For comparison, *F. prausnitzii* was similarly cultured by further adding autoclaved *E. gracilis* (9 g) in deionised distilled water (1 L).

### Real time PCR analysis

Real time PCR was performed to quantify total bacteria, using the LightCycler 96 system (Roche, Basal, Switzerland) with a universal primer set (5′-ACTCCTACGGGAGGCAGCAGT-3′ and 5′-GTATTACCGCGGCTGCTGGCAC-3′)^31^. PCR amplification was performed as per the methods described previously^18^.

### Study design for *Euglena gracilis* ingestion

This study was conducted from September 2017 to December 2017. Selected subjects were healthy (i.e., not undergoing any treatment for ailments) individuals between the ages of 40 and 60 years. The purpose and content of the study were explained thoroughly via written and oral explanation, and 28 individuals who agreed to participate and provided written informed consent were enrolled as the target population for analysis. The study protocol was approved by the Japan Conference of Clinical Research (Approval Date: 16 October 2016, Approval No. 183).

The test was designed and performed as an open study. Each faecal sample collected on day 0 was used as the baseline, and thereafter, faecal samples were collected, and the frequency of defecation and volume of faecal samples was evaluated with reference to the Bristol Stool Form Scale^19^. After day 0, each participant ingested *E. gracilis* (2 g/day) in capsule form and were requested to continuously ingest the same amount each day for 30 days. Faecal samples were collected on 14 and 30 days of ingestion.

DNA was extracted from faecal samples as previously described^32^. The 16S rRNA sequencing using the MiSeq system (Illumina, San Diego, CA, USA) was performed as previously described^32^. The V3–V4 hypervariable regions of 16S rRNA were amplified from microbial genomic DNA by PCR using bacterial universal primers (341f^33^/806r^34^) and the dual-index method^35^. Barcoded amplicons were sequenced using the paired-end 2 × 284-bp cycle of the MiSeq system using the MiSeq Reagent Kit v. 3 (600 Cycle) chemistry.

The overlapping paired-end sequencing reads were merged using the fastq-join programme with default settings^36^. Reads were processed using quality and chimera filtering as follows. Reads with quality value score of 20 for more than 99% of the sequence were extracted; chimeric sequences were removed using USEARCH v. 6.1^37^. Nonchimeric reads were submitted for 16S rDNA-based taxonomic analysis using the Ribosomal Database Project v. 2.11 and the TechnoSuruga Lab Microbial Identification database DB-BA10.0 (TechnoSuruga Laboratory, Shizuoka, Japan)^38,39^.

### Statistical analysis

Data were compared between groups using the Wilcoxon signed rank test, Mann–Whitney *U* test, two-tailed Student’s *t*-test, or Kruskal–Wallis test using JMP v. 12 (SAS Institute, Cary, NC, USA) or R v. 3.4.1 (The R Foundation, Vienna, Austria). Results with *P* < 0.05 were considered statistically significant.

## Supplementary Information

Supplementary Tables.

## Data Availability

The raw sequencing data generated in this in vivo study were deposited into the DDBJ, EMBL, and Genbank databases (http://getentry.ddbj.nig.ac.jp/) under the PSUB ID:PSUB013826; the data referring to the in vitro study were deposited into the MG-RAST server^40^ (http://metagenomics.anl.gov) in a file named ‘Model Culture System of Human Colonic Microbiota_Euglena’ under the accession numbers mgm4908075.3–mgm4908118.3.
