# Supplementary material for: The alga Euglena gracilis stimulates Faecalibacterium in the gut and contributes to increased defecation
Source: Sci Rep. 2021 Jan 13;11:1074. doi: 10.1038/s41598-020-80306-0 (PMC7806897; doi:10.1038/s41598-020-80306-0)
Supplement: Supplementary file 1 — Supplementary Tables. [file 41598_2020_80306_MOESM1_ESM.docx]

The alga *Euglena gracilis* stimulates *Faecalibacterium* in the gut and contributes to increased defecation

Ayaka Nakashima ^1*^, Kengo Sasaki ^2*^, Daisuke Sasaki ^2^, Kosuke Yasuda ^1^, Kengo Suzuki ^1^, Akihiko Kondo ^2,3^

1 The Research and Development Department, euglena Co., Ltd., Tokyo 108-0014, Japan

2 Graduate School of Science, Technology, and Innovation, Kobe University, Hyogo 657-8501, Japan

3 RIKEN Center for Sustainable Resource Science, 1-7-22 Suehiro-cho, Tsurumi-ku, Yokohama, Kanagawa 230-0045, Japan

*Corresponding author: nakashima@euglena.jp (AN), sikengo@people.kobe-u.ac.jp (KS)

**Table S1** Summary of bacterial 16S rRNA gene sequencing data.

|  |  | *In vitro* human colonic microbiota model | | |
| --- | --- | --- | --- | --- |
|  | Feces  (n = 11) | Control  (n = 11) | + Euglena  (n = 11) | + Paramylon  (n = 11) |
| Read counts | 201,687 ± 29,344 | 206,975 ± 31,373 | 190,314 ± 20,531 | 191,882 ± 36,220 |
| Observed OTUs | 1651 ± 396 | 1571 ± 352 | 1631 ± 308 | 1526 ± 269 |
| Chao 1 | 3787 ± 972 | 3824 ± 724 | 4001 ± 540 | 3871 ± 466 |
| Shannon | 6.13 ± 0.41 | 5.59 ± 0.47* | 5.76 ± 0.41 | 5.59 ± 0.35 |
| Inverse Simpson | 1.04 ± 0.01 | 1.07 ± 0.03 | 1.06 ± 0.03 | 1.07 ± 0.03 |

Eleven original fecal inoculums (Feces), corresponding to *in vitro* human colonic microbiota models (Control), corresponding models with *Euglena gracilis* (+ Euglena), and corresponding models with paramylon (+ Paramylon) were analyzed. The values show the mean ± standard deviation. Statistical differences between the Feces and Control, and each model (Control, + Euglena, + Paramylon) were evaluated. An asterisk (*) indicates a significant difference between Feces and Control samples (*P* = 0.02, Mann-Whitney *U* test).

OTUs, operational taxonomic units

**Table S2** Summary of bacterial 16S rRNA gene sequencing data.

|  | Human colonic microbiota | | |
| --- | --- | --- | --- |
|  | 0 days  (n = 28) | 14 days  (n = 28) | 30 days  (n = 28) |
| Read counts | 34,736 ± 5,331 | 36,401 ± 2,790 | 37,699 ± 4,674 |
| Observed OTUs | 153 ± 51 | 160 ± 50 | 157 ± 58 |
| Chao 1 | 154 ± 51 | 160 ± 50 | 158 ± 58 |
| Shannon | 5.59 ± 0.49 | 5.55 ± 0.53 | 5.61 ± 0.53 |
| Inverse Simpson | 0.96 ± 0.02 | 0.95 ± 0.02 | 0.96 ± 0.02 |

Fecal samples of the 28 human participants were collected before (0 days), and 14, and 30 days after the beginning of the ingestion of *E. gracilis* (2 g/day), for sequencing analysis. The values show the mean ± standard deviation. Statistical differences between day 0 and day 14 or 30 were evaluated; no significant differences were detected.

OTUs, operational taxonomic units
